# A mixed-method study of the efficacy of physical activity consultation as an adjunct to standard smoking cessation treatment among male smokers in Malaysia

**DOI:** 10.1186/s40064-016-3675-2

**Published:** 2016-11-25

**Authors:** Yuin Yi Lee, Selina Khoo, Tony Morris, Clare Hanlon, Lei-Hum Wee, Eng Wah Teo, Yuhanis Adnan

**Affiliations:** 1 Sports Centre, University of Malaya, Kuala Lumpur, Malaysia; 2 Institute of Sport, Exercise and Active Living (ISEAL), College of Sport and Exercise Science, Victoria University, Melbourne, Australia; 3 Faculty of Health Sciences, National University of Malaysia, Kuala Lumpur, Malaysia

**Keywords:** Smoking intervention, Case study, Motivational interviewing, Tobacco use, Behavioral treatment

## Abstract

**Background:**

This study examined the effectiveness of using Physical Activity Consultation (PAC) as an addition to the standard smoking cessation treatment in Malaysia. We explored participants’ experiences in terms of physical activity and smoking abstinence with the combined PAC and smoking cessation intervention.

**Methods:**

Walk-in smokers from a local smoking cessation clinic volunteered for the 8-week intervention program, while undergoing standard smoking cessation treatment. In Week 1, a facilitator conducted a face-to-face intervention to explore participants’ involvement in physical activity and helped to set physical activity strategies and goals for participants to increase physical activity levels. Participants were provided with follow-up phone calls at Weeks 3 and 6. Participants answered questionnaires that measured smoking withdrawal (Shiffman–Jarvik Withdrawal Scale), cessation self-efficacy (Cessation Self-efficacy Questionnaire), physical activity involvement (International Physical Activity Questionnaire), and mood (Brunel Mood Scale) upon recruitment, at post-intervention and at follow-up 3 months after the intervention ended. Participants also responded to interviews about their experiences with the PAC and smoking cessation treatment at post-intervention and at 3-month follow-up.

**Results:**

Seven participants completed the program until follow-up. All were successfully abstinent. Only two participants increased physical activity levels, whereas others maintained their physical activity levels or showed slight decreases. Several themes were identified in this study, including participants’ experiences with withdrawal symptoms, smoking cessation self-efficacy, triggers to smoking cessation, thoughts on standard smoking cessation treatment in Malaysia, physical activity involvement, mood, and thoughts and beliefs on combining smoking cessation and physical activity.

**Conclusions:**

This study suggests PAC was helpful in maintaining or increasing the overall physical activity levels of participants and could assist with smoking abstinence. Findings showed that all participants who stayed in the program were successfully abstinent. In general, the findings in this study provided promising results for further research on PAC as an adjunct to smoking cessation treatments in Malaysia.

*Trial registration* This intervention is registered with the Australian New Zealand Clinical Trials Registry (Trial registration number: ACTRN12616000269437).

**Electronic supplementary material:**

The online version of this article (doi:10.1186/s40064-016-3675-2) contains supplementary material, which is available to authorized users.

## Background

With approximately 1.1 billion smokers worldwide today, smoking remains a global concern (Jha 2009). The World Health Organization (2012) reported that tobacco use is responsible for 12% of all deaths among adults aged 30 years and over, making it one of the leading risk factors for mortality globally. As in many other countries, smoking is a major concern in Malaysia; 22.8% of all adults smoked in 2015 (Institute for Public Health 2015). The Institute for Public Health (2015) reported that the smoking prevalence of current tobacco smokers (which include daily smokers, occasional smokers, and all smokeless tobacco users) in 2015 was higher among Malaysian adult males (43.0%) compared to adult females (1.4%). The prevalence among Malaysian adults had increased from 2011 to 2015, with 19.3% of adults smoking in 2011. Researchers have reported that Malaysian smokers were well aware of the detrimental effects of smoking, but many still continued to smoke (Institute for Public Health 2012).

In an effort to reduce the number of smokers in Malaysia, several quit smoking programs were initiated in Malaysia, such as “Tak Nak!” or “Say No!”, a nationwide anti-smoking campaign that used mass and print media to spread public awareness regarding the dangers of smoking (Hong et al. 2013). Other efforts include “Kempen Nafas Baru Bermula Ramadhan” (“New Breath Beginning Ramadhan”) (Ministry of Health Malaysia 2014), quit smoking clinics nationwide (Ministry of Health Malaysia 2010), and quit line services over the phone. In order to further enhance smoking cessation efforts, it is important to study smoking behavior of Malaysians, as well as related factors, such as withdrawal, mood, and self-efficacy that could contribute to effective smoking cessation programs. It is difficult for smokers to stop smoking because of the addictive nature of cigarette smoking (National Institute of Drug Abuse 2012). Past studies in Malaysia indicated no standard treatment strategies at the quit smoking clinics nationwide. They also indicated there were differences in the success rate between clinics (Wee et al. 2011). Wee et al. (2011) found high default rates of 51.8% at 6-month follow-up. Research is needed to examine how different approaches to behavioral support, skills of practitioners and availability of pharmaceutical support affect smoking cessation success rates. One behavioral technique that has shown promise is promoting physical activity (PA) participation as an adjunct to smoking cessation (e.g. Taylor et al. 2014). In this study, we examined the impact of Physical Activity Consultation (PAC) as an adjunct to standard smoking cessation treatment among males in Malaysia.

Previous literature has suggested several factors that may predict relapse, such as mood, withdrawal symptoms, and self-efficacy. Negative mood has been associated with barriers to smoking cessation (Vangeli et al. 2010; Zhou et al. 2009), whereas expectations of positive mood outcomes derived from smoking, such as mood enhancement and stress relief, have been linked to motivations to initiate or continue smoking (Byrne and Mazanov 2003; McKenna et al. 2003). According to Bernard et al. (2013), those who experience depression are often less successful in smoking cessation and tend to experience elevated and prolonged levels of withdrawal symptoms. Smokers with high baseline depressive symptoms may develop low self-efficacy for smoking cessation which will increase the risk of relapse (Cinciripini et al. 2003). Withdrawal symptoms have also been reported to predict smoking cessation outcomes. Allen et al. (2008) found that prior to relapse, withdrawal symptoms increased and peaked on the day of relapse. After relapse, withdrawal symptoms were found to decrease rapidly. Researchers have also associated the urge to smoke with lower self-efficacy to quit smoking and feelings of anxiety (Niaura et al. 2002). Self-efficacy refers to people’s belief in their ability to successfully perform a behavior to achieve certain outcomes (Bandura 1977). Studies have shown that higher levels of smoking cessation self-efficacy predict higher likelihood of smoking cessation and maintenance of abstinence (Morrell et al. 2011; Perkins et al. 2012).

PA could help to remove or reduce barriers to smoking cessation and factors associated with relapse, such as withdrawal, negative affect and low self-efficacy (Abrantes et al. 2009; Horn et al. 2013). Studies have suggested that PA can help to reduce cravings during cessation, and those who exercised reported higher continuous abstinence compared to those who were not active (Haasova et al. 2013; Taylor et al. 2007). PA could also reinforce a healthier lifestyle which does not include smoking, and this might help to enhance cessation self-efficacy (Prochaska et al. 2008). Success in achieving PA goals can also increase self-efficacy in achieving other goals, such as smoking cessation (Abrantes et al. 2009; Horn et al. 2013). While a number of studies have used PA to improve smoking cessation results, most of these studies used exercise prescription methods (Harper et al. 2012; Horn et al. 2013). Dale et al. (2009) who prescribed PA to participants found that 70% of the participants did not maintain their PA after the prescription was removed. Many of the participants explained that they did not continue with the exercise because the supervision was removed (Dale et al. 2009). Stuntz and Weiss (2010) explained that it is important for participants to feel that they make their own decisions and personal choices in order to predict long-term PA participation. In a more recent study, Taylor et al. (2014) explored the efficacy of using a client-centered intervention, Exercise Assisted Reduction then Stop (EARS) in which participants set their own level of engagement in PA after having a face-to-face interview with the facilitators. Results from this study have shown that engagement in PA reduced to baseline after intervention has ended. However, Taylor et al. (2014) explained that this could be due to the high level of baseline PA of the participants. Participants who participated in this study were also given subsidized access to PA opportunities, which could explain why participants’ level of PA reduced to baseline after this access was removed at the end of the intervention.

 An intervention developed to increase PA in the general population is PAC (Loughlan and Mutrie 1996). PAC is a customized intervention that combines a number of techniques supported by research, including motivational interviewing (Miller and Rollnick 2002), self-efficacy theory, including mastery goal setting (Bandura 1977), the Transtheoretical Model of Behavior Change (Prochaska and DiClemente 1982) and relapse prevention (Marlatt and Gordon 1985). In a meta-analysis by Gourlan et al. (2016), theory-based interventions, such as the Transtheoretical Model, are shown to be effective in promoting PA in workers as well as various healthy or unhealthy adults. Studies on the use of PAC to promote PA have shown the effectiveness of the approach. Kirk et al. (2001) gave exercise consultations to people with Type 2 diabetes, which included discussion on past and present physical activities, benefits and obstacles to becoming more active, goal setting, social support and relapse prevention. They concluded that exercise consultation was more effective than standardized exercise leaflets in promoting short-term exercise behavior. In another study, Maddison et al. (2014) used exercise counselling to help enhance smoking cessation outcomes. They found that those who received exercise counselling increased their levels of leisure PA, compared to those who did not receive exercise counselling.

In the present study, we examined the effectiveness of PAC as an adjunct to the standard smoking cessation treatment used in Malaysia. In particular, using in-depth interviews supported by tests of withdrawal, cessation self-efficacy, PA, and mood, to explore each participant’s subjective experiences of the combination of PAC and standard smoking cessation.

## Method

### Participants

Participants were recruited from a government smoking cessation clinic located in Kuala Lumpur. This clinic was chosen based on the large number of walk-in participants. It was the most active clinic in Malaysia with a regular smoking cessation program. In this study, only male participants were included due to the demographics of smokers in Malaysia where close to half adult males smoke compared to less than 2% of females (Institute for Public Health 2015). Participants were screened to ensure that they met our inclusion criteria: males, over 18 years of age, smoked 10 or more cigarettes per day, the first cigarette smoked within 30 min of waking, and were not medically dependent or intellectually impaired. The cut-off of 10 cigarettes was to ensure that smokers were moderate or heavy smokers (Clair et al. 2011) and smoking within 30 min of waking is a reliable indicator of nicotine dependence (Zwar et al. 2014).

### Measures

Upon recruitment, participants completed a Demographic Form and four questionnaires in the Malay language.

#### Demographic Form

The demographic form covered information on age, occupation, smoking history, current smoking habits, previous smoking cessation attempts, reasons for quitting smoking, and participation in PA.

#### Shiffman–Jarvik Withdrawal Scale (SJWS; Shiffman and Jarvik 1976)

The SJWS is a 25-item questionnaire that measures participants’ withdrawal symptoms and desire to smoke. The SJWS consists of five subscales: craving, psychological symptoms, physical symptoms, sedation, and appetite. The translated Malay version of this questionnaire (SJWS-M) had an overall Cronbach’s alpha, *α* = .66, with three of its subscales (cravings, physical, psychological symptoms) showing alpha values greater than .70 (Teo et al. 2015). It also showed good test–retest reliability (*r* = .76) (Teo et al. 2015).

#### Cessation Self-efficacy Questionnaire (CSEQ; DiClemente 1981)

The CSEQ measures participants’ beliefs in their capability to avoid smoking in various situations. Pearson item-scale correlations were reported at an average of .68 ranging from .58 to .76 (DiClemente 1981). Internal consistency was good with an α range of .87–.96 (DiClemente et al. 1991; Prapavessis et al. 2007). The translated Malay version of this questionnaire (CSEQ-M) also demonstrated good internal consistency with Cronbach’s alpha of .90 and good test–retest reliability (*r* = .80) over 2 weeks (Teo et al. 2015).

#### International Physical Activity Questionnaire (IPAQ; Craig et al. 2003)

In this study, we used the 7-item short form IPAQ. Craig et al. (2003) reported good reliability for the IPAQ with *r* > .7. This questionnaire was translated to Malay language by Chu and Moy (2015) and was tested for reliability and validity among the Malaysian population. The Malay version of the IPAQ (IPAQ-M) showed good reliability with intra-class correlation coefficients (ICC) of .84–.92, except for walking (ICC = .54), which was still considered moderately correlated. The IPAQ-M also showed good validity with Cohen’s coefficient, κ = .89 (Chu and Moy 2015). The IPAQ measures and records the amount of PA of participants in four intensity levels: vigorous intensity, moderate intensity, walking, and sitting. The amount of PA is based on the number of days and minutes per day of doing each level of PA in the past 7 days. Raw scores are then converted into estimated metabolic equivalents (METs). METs are used to calculate MET-minutes/week scores.

#### Brunel Mood Scale (BRUMS; Terry et al. 1999)

The BRUMS records participants’ moods states. The six mood states measured are: tension, depression, anger, fatigue, vigour, and confusion. The BRUMS is a valid and reliable measure with α values for all subscales above the recommended value of .70 (Lan et al. 2012; Terry et al. 1999, 2003). The BRUMS was translated to Malay language by Hashim et al. (2010) and was tested for reliability and validity among the Malaysian population. The BRUMS-M showed good factorial validity and satisfactory reliability. Alpha coefficients were reported to range from .58 to .73 (Hashim et al. 2010).

#### Interviews

Two interviews were conducted, a post-test interview at Week 9, after the PAC intervention ended, and a follow-up interview at Week 21, after 12 weeks with no intervention support. At post-test (Week 9), participants discussed their smoking history, smoking cessation experience, smoking cessation status, opinions on smoking before and after the cessation experience, PA involvement and opinions on PA before and after the intervention. Topics of discussion also focused on thoughts and opinions regarding the relationship between PA and smoking cessation. Questions were conversational and flexible based on the information provided by participants. At follow-up (Week 21), we interviewed participants about their experiences and feelings since the intervention ended. We also asked them about progress of their smoking cessation and PA after the intervention.

### Intervention

The intervention comprised a combination of the standard Malaysian smoking cessation treatment and PAC to promote PA.

#### Standard smoking cessation treatment

Smoking cessation consultation sessions were provided by a nurse with a counselling licence approved by the Malaysian Board of Counsellors. The nurse gave the participants behavioral counselling. Nicotine Replacement Therapy (NRT) was provided as well in the form of varenicline (Champix). Participants attended the clinic after 1 week for follow-up. Subsequent visits were scheduled by the clinic nurse depending on participants’ needs. These consultation sessions were conducted as often as needed by the participants until they were successfully abstinent. We arranged for the clinic nurse to organise one visit specifically at Week 9 and one at Week 21 for the purpose of the interviews.

#### Physical activity consultation (PAC)

Motivational interviewing (Miller and Rollnick 2002) is the primary consultation style used in the PAC. It is often described as a way of being with people and promoting a safe and encouraging environment for therapists to help clients’ growth and change (Westra and Aviram 2013). Motivational interviewing focuses on freedom and autonomy of clients in their change process. Another core component of the PAC is matching support to individuals’ stage of change, based on the Transtheoretical Model of Change (Prochaska and DiClemente 1982). This model proposes five stages of change: pre-contemplation, contemplation, preparation, action and maintenance (Marcus et al. 1992). The PAC also uses mastery goal setting, which focuses on Bandura’s (1977) concept of self-efficacy and involves setting step-by-step, achievable goals in order to increase experiences of mastery that enhance self-efficacy. Lastly, the PAC incorporates relapse prevention (Marlatt and Gordon 1985) into the intervention. Participants are prepared for the chance of relapse and guided on effective courses of action in the event of such relapse. Participants are also advised that relapse does not mean failure, but could be a trigger for success.

PAC was conducted by a facilitator who maintained contact with participants for 21 weeks. PAC consisted of one face-to-face consultation session and two follow-up phone sessions. In the face-to-face consultation, we sought to understand participants’ level of PA, general view on PA, provide motivation and help them shape their own path for change, at the same time providing them with a sense of autonomy. We assessed participants for their current stage of change and advised on PA to suit their current fitness levels. Advice also covered realistic goal setting and relapse prevention. Phone calls at Weeks 3 and 6 functioned as follow-ups with participants on their progress, as well as providing encouragement and motivation. In Weeks 0, 3 and 6 when the facilitator communicated with the participants, the intervention included all the behavior change techniques of PAC, described in the previous paragraph. These techniques were administered as appropriate to address the presentation of progress and concerns by each participant (Additional file 1).

### Procedure

#### Recruitment

Recruitment was conducted from 15 April 2013 to 15 January 2014. Smokers who attended the clinic for the first time to seek smoking cessation treatment were invited to participate in the study. During the first visit, the nurse provided smoking cessation consultation and scheduled a follow-up meeting a week later. After the smoking cessation consultation during the follow-up meeting, the nurse explained the study to participants and those who were interested to participate met the first author. The first author, who is a Psychology graduate with training and experience conducting questionnaire and interview studies, then explained the study in more detail and those who agreed to participate provided written consent. Acting as facilitator, the first author administered all the questionnaires, carried out all the interviews and executed all aspects of the intervention, following training in PAC delivered by an author with considerable experience of conducting PAC interventions.

#### Intervention Phase

At *Week 0*, we briefed participants about the nature of the study and informed them that their participation in the study was voluntary and they could withdraw at any time. Participants completed the demographic form, the SJWS-M, IPAQ-M, BMS-M, and CSEQ-M. The facilitator then conducted a face-to-face PAC session that lasted between 30 and 40 min. The face-to-face intervention was conducted in a quiet room in the clinic. We advised participants to commence their PA as agreed with the facilitator. During *Weeks 1 to 2* participants implemented the PA that they had agreed in the face-to-face intervention without contact with the facilitator. At *Week 3* the facilitator followed up participants with a phone interview at a time convenient to the participants. The phone interview lasted between 15 and 20 min and focused on any concerns participants had about their PA, reinforcement of goals they had set for themselves and general encouragement. During *Weeks 4 and 5* participants continued to perform PA. At *Week 6* the facilitator contacted participants by telephone, conducting an interview similar in format to the Week 3 interview. During *Weeks 7 and 8* participants continued their self-selected PA and the intervention terminated at the end of Week 8.

#### Post-intervention data collection

**Fig. 1:**
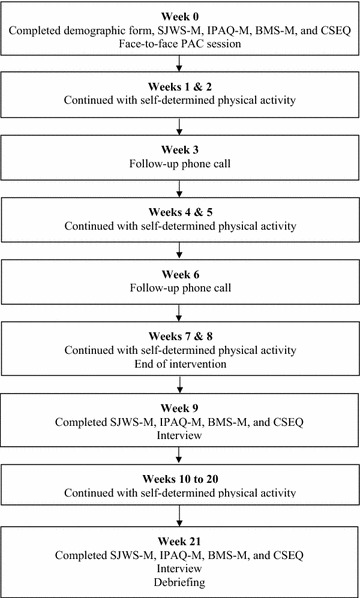
Flow of the study

At *Week 9*, participants returned to the clinic for another smoking cessation consultation session. Following this consultation, the facilitator administered the same set of questionnaires (SJWS-M, IPAQ-M, BMS-M, CSEQ-M). Then she conducted a post-test interview. During *Weeks 10–20* participants continued to perform their PA with no contact from the facilitator. At follow-up in *Week 21*, the facilitator administered the four questionnaires (SJWS-M, IPAQ-M, BMS-M, CSEQ-M) and interviewed participants about their experiences and feelings since the intervention ended. We then debriefed and thanked participants for their involvement. Figure 1 displays the flow of the study.

### Data analysis

Data from the questionnaires were analyzed using SPSS (Version 20.0). We used QSR NVivo (Version 10.0) to analyze qualitative information from the interviews. We sought an essentialist or realist type of knowledge, which reports the experiences, meaning and reality of the participants. We performed an inductive thematic analysis to identify and report patterns of themes within the data. According to Braun and Clarke (2006), thematic analysis is not only an appropriate technique to identify patterns within data, but also efficient in helping to organize and describe a data set in rich detail by using an inductive approach. Only general patterns are reported here.

## Results

Seven participants continued until Week 21, completing the questionnaires and the follow-up interviews. They were aged between 24 and 54 years and have been smoking for 3–39 years. They showed overall decrease in withdrawal symptoms and increased smoking cessation self-efficacy. The majority of our participants cited health as a trigger to smoking cessation. Participants also discussed their thoughts on the standard smoking cessation treatment in Malaysia and their experiences with the treatment. In addition to that, participants shared their experiences and involvements in PA throughout the program. Lastly, our data also reflected participants’ mood as well as their personal thoughts and beliefs on smoking cessation and PA. The results presented integrate data from the questionnaires and interview responses.

### Withdrawal symptoms

During the interviews, only one participant expressed withdrawal symptoms during early stages of smoking cessation. However, these symptoms diminished over time and by Week 9, all participants were not experiencing any withdrawal symptoms. Consistent with this, results from the SJWS-M indicated that participants showed an overall decrease in withdrawal symptoms throughout the program. Comparing the means, there was a decrease in every subscale (cravings, psychological symptoms, physical symptoms, and sedation) except appetite. The results of the SJWS-M are shown in Table 1.

**Table 1:** SJWS-M and CSEQ-M Scores for Week 0, Week 9 and Week 21 (n = 7)

Questionnaires	Week 0		Week 9		Week 21	
	Mean	Standard deviation	Mean	Standard deviation	Mean	Standard deviation
SJWS-M						
Craving	13.67	5.01	9.71	5.50	6.71	2.21
Physical	7.17	2.99	7.00	2.08	7.00	3.27
Sedation	3.17	2.14	1.57	1.13	1.71	1.11
Psychological	14.33	5.50	10.43	3.10	10.57	2.99
Appetite	4.00	2.10	4.00	2.00	4.00	1.83
Global score	42.33	11.86	32.71	8.24	30.00	5.86
CSEQ-M						
Total score	65.71	18.68	72.71	22.40	77.29	8.96

### Self-efficacy for smoking cessation

During the interviews, all participants stated that they were completely abstinent by Week 9 of the program, and reported high levels of confidence in maintaining smoking abstinence at both Week 9 and Week 21. Even though one participant mentioned the urge to smoke when being out with smoker friends, all participants showed no hesitation in stating their confidence about maintaining abstinence in the future. Results (Table 1) from the CSEQ-M indicated that there was an increase in smoking cessation self-efficacy consistent with participants’ interview reports.

### Triggers to smoking cessation

All the participants cited ill health as a reason to quit smoking. Many reported decreased stamina and lung capacity due to smoking and said they would like to improve that by quitting smoking. According to one participant,

When I went to a conference, I was given a chance to test my lung capacity of my lung age. I was not very happy already, because, you know, I used to be a school athlete, and now my lungs are not really that healthy, so that’s why I actually want to stop smoking. (*S1, 26* *years, 15 cigarettes a day, started smoking at the age of 18*)

Those who participated in sports expressed their desire to enhance performance by quitting smoking. One participant explained that smoking reduces stamina and those who want to excel in sports would be motivated to reduce smoking. Although the price of cigarettes has been increasing in Malaysia, only one participant mentioned this as a reason for smoking cessation.

### Thoughts on the standard smoking cessation treatment in Malaysia

Participants reported that they were extremely pleased with the services and help provided while undergoing the smoking cessation treatment. Many participants acknowledged the efficacy of the treatment and named it as a major contributor to their success in smoking cessation. Participants were provided with ongoing support throughout their cessation period and even after achieving abstinence. However, participants also observed that public awareness of smoking cessation treatment is low. Participants reported that information regarding access to smoking cessation treatment in Malaysia was hard to obtain and suggested activities to increase awareness through social and print media.

### Physical activity involvement

Common physical activities were jogging, walking, and futsal. During the interviews, two participants stated that they had increased their PA. Most participants reported maintaining PA, although they expressed an intention to increase PA. Obstacles that were commonly faced for participating in PA were lack of friends for team sports (e.g., futsal), lack of time, and deteriorating health. One participant (*S2, 26* *years, 10 cigarettes a day, started smoking at the age of 15*) explained, “I like sports. Futsal. Before this I played, but we need to have friends to participate in a game like futsal. Ah, so now friends are no longer [around]”. Those who successfully increased PA were younger participants in their early twenties. Factors that contributed to their success were the availability of exercise facilities and friends to participate with. According to one participant (*S1, 26* *years, 15 cigarettes a day, started smoking at the age of 18*), “Whenever I like, after gym maybe I go for swimming perhaps. Or sometimes, after badminton I go swimming. Because it’s in my friend’s condo, so everything is very convenient.” Reasons for participating in PA were discussed. The main reason given was health benefits. All participants had experienced some form of deterioration in terms of health and stamina due to smoking and were aware of the benefits of exercising. According to one participant (*S3, 31* *years, 20 cigarettes a day, started smoking at the age of 15*), “PA for me, it’s good. One, it’s for health. Another it’s for us to interact with people, our friends.”

The IPAQ-M results in Table 2 showed participants increased their moderate intensity of PA. Participants reported that their walking and vigorous intensity activities increased from Week 0 to Week 21. There was an increase in moderate intensity PA at Week 9, but it decreased at Week 21. However, the scores for moderate intensity exercise increased from Week 0 to Week 21. The total MET-minutes/week scores also increased throughout the program, indicating that participants increased their overall PA.

**Table 2:** IPAQ-M Scores for Week 0, Week 9 and Week 21 (n = 7)

Questionnaires	Week 0		Week 9		Week 21	
	Mean	Standard deviation	Mean	Standard deviation	Mean	Standard deviation
IPAQ-M (MET-minutes/week)						
Walking	346.50	663.50	1503.86	3045.23	1885.71	2139.95
Moderate	257.14	201.14	411.43	587.29	354.29	302.37
Vigorous	274.26	544.27	651.43	611.32	782.86	857.24
Total	877.93	1235.81	2566.71	3694.96	3526.67	3005.45

### Self-efficacy for physical activity

During the interviews at Week 9, only one participant stated that he was not confident about maintaining or increasing PA in the future due to his lack of knowledge about PA. The other six participants were confident in increasing or maintaining PA. At Week 21, a participant expressed uncertainty in performing PA due to health problems, while another had no intention to increase PA. Five participants expressed confidence in increasing or maintaining PA at Week 21.

### Mood

Based on behavioral observations during the interviews, we observed relatively stable mood at Week 9 and Week 21. Language used by participants, tone of voice as they talked in interviews and limited physical animation throughout the interviews reflected mood that was neither very positive nor notably negative. This is consistent with the mean scores for the BRUMS-M throughout the program (Table 3). In general, there was a slight decrease in the scores for anger, confusion, depression, and fatigue at Week 9 compared to baseline, and the scores increased minimally from Week 9 to Week 21. The scores for tension remained the same between baseline and Week 9 and then increased slightly at Week 21. On the other hand, a slight decrease was observed in vigour scores from baseline to Week 21. Overall, observations during the interviews and BRUMS-M results indicated that mood was relatively stable and moderate throughout the study.

**Table 3:** BRUMS-M Scores for Week 0, Week 9 and Week 21 (n = 7)

BRUMS-M	Week 0		Week 9		Week 21	
	Mean	Standard deviation	Mean	Standard deviation	Mean	Standard deviation
Anger	52.00	7.92	45.57	2.82	47.29	8.69
Confusion	46.86	5.11	43.29	3.40	47.00	9.85
Depression	46.57	4.65	44.14	1.95	46.43	9.07
Fatigue	45.57	7.14	40.29	1.38	43.43	5.77
Tension	44.57	4.54	44.57	3.36	45.29	8.69
Vigour	61.00	6.71	60.86	4.02	60.05	8.67

### Combining smoking cessation and physical activity

In general, all of our participants were successful in the smoking cessation aspect of this study, while most of our participants reported increases in PA (i.e., increased total MET-minutes/week scores).

During the interviews we asked participants to reflect on their experiences of undertaking a program that combined a standard smoking cessation treatment with a PA intervention, PAC. Participants reflected on the impact of smoking cessation on PA and the role of PA as an adjunct to smoking cessation.

### Smoking cessation enhances PA involvement

Participants indicated that their PA had improved after quitting smoking. Breathlessness had reduced and participants felt that they could perform PA more comfortably.

But now–It’s not really that easy to get breathlessness when I play badminton, when I go for swimming, when I do my jogs. Compared to before, yeah. Definitely. Because last time I remember, I went to gym, after cycling for like 15 min, 20 min, ah okay, now I need a smoke. I need a smoke. So, now it doesn’t anymore. *(S1, 26* *years, 15 cigarettes a day, started smoking at the age of 18)*

Most of the participants reported increased stamina and less fatigue after smoking cessation. Activities such as jogging could be performed for longer periods of time.

#### PA helps with smoking cessation

Four of the seven participants stated that increasing PA could help with smoking cessation. A participant proposed that PA could help people to overcome cravings and urges to smoke. According to one of our participants, PA helped him to feel healthier and this led him to stay away from smoking.

Because I feel that every time I work out, I tend to be a bit more healthy. So I tend not to want to smoke. You know. So… Yeah. Yeah. Because if I were to smoke, then it would just waste my effort of working out. (*S1, 26* *years, 15 cigarettes a day, started smoking at the age of 18*)

## Discussion

In this research, we explored the strategy of using a PA intervention to help smokers in a standard smoking cessation program to stop smoking or reduce smoking. In this study, participants discussed their experience of smoking cessation, PA, and continuing both behaviors throughout the program and during the 3 months of follow-up without support. The quantitative data was highly variable for the sample size so we did not conduct inferential statistics. We consider that the means for the questionnaires reported in the results are not meaningful reflections of a consistent pattern. Therefore, we do not discuss the quantitative data further. We observed several themes that emerged in the qualitative data, including health as a trigger for smoking cessation, free nicotine replacement therapy, the benefit of smoking cessation for performing PA, and the importance of social support for PA participation.

Overall, the seven participants who completed questionnaires at pre-intervention, post-intervention and follow-up and interviews in Weeks 9 and 21 were successfully abstinent by Week 9 and continued to be abstinent at Week 21. By Week 9, interview results indicated that none of the participants reported any withdrawal symptoms or difficulties maintaining abstinence. Participants’ responses to the questionnaires reflected similar patterns, that is, withdrawal symptoms decreased and self-efficacy for abstinence increased. A strong theme that emerged among the participants was health as a trigger for smoking cessation. All seven participants wanted to improve their health, which they perceived to be deteriorating due to smoking. They reported that smoking caused shortness of breath, decreased stamina, increased sickness frequency, and decreased lung capacity. This is supported by previous studies (Martinson et al. 2003; Twardella et al. 2006), which suggested that experiencing health consequences of smoking led to efforts to stop smoking. Based on this finding we propose that researchers should explore the opportunities for marketing campaigns to target health as a primary reason to cease smoking.

All seven participants provided positive comments regarding the efficacy of the smoking cessation treatment. They thought that it was helpful that the government provided free nicotine replacement therapy to the public. According to the participants, having nicotine replacement therapy through the smoking cessation treatment was a major contributor to their success in smoking cessation. This finding is supported by a review by Silagy et al. (2000), who reported the effectiveness of using nicotine replacement therapy in smoking cessation. After-hours support was also provided to the participants as they were told that they could visit or telephone the center at any time for advice about smoking cessation. Good rapport was established between the nurse who provided counseling and the participants. This reinforces the importance of social support in smoking cessation interventions (Carlson et al. 2002; Wagner et al. 2004). However, participants reported that public awareness regarding smoking cessation treatments in Malaysia was very low. In our search for publicity on smoking cessation treatment we also found minimal publicity. This warrants greater efforts on raising awareness of the public regarding the availability of smoking cessation treatments in Malaysia and the free nicotine replacement therapy and social support that is available.

In terms of PA, two participants stated during the interviews that they increased PA levels, while others showed intentions to do so in the future. On the other hand, IPAQ results suggested that participants substantially increased PA throughout the program. This discrepancy needs to be further explored. Participants also reported that as they all abstained from smoking, their subjective experience was that PA became easier, for example, due to improved breathing. Perhaps they were doing PA for longer durations or at higher intensity, but subjectively felt more comfortable so they did not perceive that they had increased their PA. Walking or jogging was a popular PA among the participants. These activities are flexible, inexpensive and do not require a partner or a team. Such activities could easily fit into participants’ schedules. We observed that younger participants enjoyed PA that involved participation with friends (team sports, such as futsal or badminton), whereas older participants preferred to participate in PA that could be easily done on their own in their own homes or in familiar venues. However, even those who enjoyed sports with friends explained that it was not always easy to find friends with whom to participate in sports, and they often turned to walking or jogging as a form of exercise. Findings from this study indicate potential for future smoking cessation programs to link with leisure centers, so that participants can select from a variety of PA programs and be more involved in a social network.

Our research findings suggest that smoking cessation could help to enhance PA. This also supports studies that were previously conducted on the effects of smoking cessation on exercise (Asthana et al. 2012). Findings also show that PA helps smoking cessation yet studies have been inconclusive on this finding (Treviño et al. 2014; Ussher et al. 2014; Williams et al. 2010). The results from our research strengthen the proposition that PA helps smoking cessation. In particular the PAC component of PA that provides information as well as social support to participants was particularly helpful. To further substantiate the proposal that PA incorporating PAC helps smoking cessation in Malaysia, research with a larger sample is required. It is important to note that the participants in this study were smokers who approached the clinic for a nicotine replacement therapy-based smoking cessation treatment. They had no initial awareness of or intention to undertake a PA intervention. The participants in this study were relatively active at the start of the study and this could have moderated the impact of PA in this study. Our findings indicate that variables, such as starting PA level and motives for undertaking the combined smoking cessation and PA intervention, should be controlled or carefully monitored.

Findings revealed that there was a lack of public awareness regarding smoking cessation treatments in Malaysia and more effort could be made to promote the availability of smoking cessation treatments in the country. Studies have shown that smokers who tried to stop smoking supported by formal smoking cessation treatments were more likely to succeed compared to those who tried to stop on their own (Marlatt et al. 1988; Zhu et al. 2000). Increasing public awareness about the smoking cessation treatments in Malaysia is important in helping more Malaysians quit smoking.

We noted several limitations in this study. First of all, we were not able to control for factors, such as participants’ backgrounds and smoking history as the participants were walk-in smokers. As with other smoking cessation research (Maddison et al. 2014), we also observed a slow recruitment rate. Another limitation of this study was that there was a high drop-out rate of participants. Although we recruited 41 participants at baseline, only seven participants continued till Week 21. It is possible that participants who were successful in smoking cessation were the ones who stayed through the program until Week 21, whereas those who dropped out were not successfully abstinent. This self-selection may have resulted in a biased sample (Heckman 1979). The sample was also limited to male participants due to the demographics of smokers in Malaysia where the majority of smokers are males (Institute for Public Health 2015). Future studies should seek to study the efficacy of PAC for both male and female smokers. Finally, it is important to acknowledge that the facilitator who conducted the intervention did not have formal training as a counsellor, which could have affected the efficacy of the PAC in this study. Although the facilitator was provided with ample training in the delivery of the PAC, perhaps delivery by a qualified counsellor would enhance the efficacy of PAC.

## Conclusion

This study provides insight into participants’ perspectives about the efficacy of PAC as an aid to smoking cessation. The findings suggested that PAC was considered effective particularly for those who increased their PA. We also gained understanding of participants’ experiences with smoking cessation in Malaysia, as well as the factors that influenced participants’ decisions regarding smoking. Findings revealed that the health factor was a main trigger for smoking cessation attempts. The insights gained from this study can help smoking cessation clinics support smokers who are planning to quit by incorporating PA into smoking cessation programs. Future smoking cessation programs can also look at factors such as motivations to quit smoking and obstacles faced during the quit smoking process when counselling smokers trying to quit smoking into smoking cessation programs.

## Additional file

**Additional file 1.** Session-by-session physical activity consultation (PAC) intervention content.

## Abbreviations

PA: physical activity
PAC: P hysical Activity Consultation
SJWS: Shiffman–Jarvik Withdrawal Scale
CSEQ: Cessation Self-efficacy Questionnaire
IPAQ: International Physical Activity Questionnaire
BRUMS: Brunel Mood Scale
