# Supplementary material for: A mixed-method study of the efficacy of physical activity consultation as an adjunct to standard smoking cessation treatment among male smokers in Malaysia
Source: Springerplus. 2016 Nov 25;5(1):2012. doi: 10.1186/s40064-016-3675-2 (PMC5122530; doi:10.1186/s40064-016-3675-2)
Supplement: Supplementary file 1 — Additional file 1. Session-by-session physical activity consultation (PAC) intervention content. [file 40064_2016_3675_MOESM1_ESM.docx]

**Supplementary File**

***Session-by-Session Physical Activity Consultation (PAC) Intervention Content***

This supplementary file describes the typical content of the three PAC sessions, which took place in Weeks 1, 3, and 6

| **Week** | **Theoretical Base** | **Description of Content** |
| --- | --- | --- |
| Week 1:  Face-to-face  Session | Motivational Interviewing | - Building of rapport through shared exploration of previous physical activity experience - Encouragement to talk through active listening and reflection - Demonstration of non-judgmental support through positive acceptance of physical activity regardless of type and level - Empowering by asking what participant thinks they can do in terms of type of activity and level or intensity - Demonstration of advisory role by suggesting rather than prescribing |
|  | Self-efficacy | - Exploring what participant thinks they can do - Discussing a goal for the first two weeks - Ensuring goal is achievable to produce mastery experience to enhance self-efficacy for chosen activity - Agreeing goal with participant in empowering language “so you are telling me that you plan to do…” |
|  | Trans-theoretical Model | - Identifying current stage of change - Providing support that corresponds to the current stage of change - For example:   - giving information about reasons to do physical activity (pre-contemplation);   - discussing costs of not changing versus benefits of changing (contemplation)   - planning requirements to do the chosen activity, including kit, equipment, facilities (preparation) |
|  | Relapse Prevention | - Discussing the difference between a lapse and relapse - Considering strategies to avoid lapses, e.g., exercising indoors if it is too hot or humid to exercise outdoors - Identifying responses to lapses in order to prevent relapse, e.g., after missing activity due to wet weather, get waterproof kit for next occasion |
| Week 3:  First  Telephone  Session | Motivational Interviewing | - Enhancing rapport by sharing experiences of first two weeks of physical activity - Encouraging discussion of issues by praising activity done, even if limited compared to goals - Exploring feelings of success and achievement to reinforce empowerment - Reflecting on problems and obstacles experienced and encouraging participant to think of ways to resolve problems and surmount obstacles - Reinforcing participant’s positive experience of autonomy in choosing and undertaking own physical activity |
|  | Self-efficacy | - Discussing experiences of physical activity during first two weeks of program - Praising level of activity attained to increase feelings of mastery - Reflecting on degree to which goals set in Week 1 were attained - Encouraging participant to revise goals based on experience   - If goals were easily attained and mastery was experienced, support goals being increased   - If goals were not attained, reflecting on whether they were too high and should be reset to suit currently achievable levels to ensure mastery |
|  | Trans-theoretical Model | - Identifying current stage of change - Praising if participant has moved in a positive direction - Reflecting if stage of change has remained unchanged - Supporting and problem-solving with participant if stage of change has moved in a negative direction - Providing stage-appropriate advice - For example, if stage has moved from preparation to action, discussing behavioral techniques to sustain activity, such as keeping a log to show progress of activity |
|  | Relapse Prevention | - Exploring any experiences of lapsing in activity during Weeks 1 to 3 - Examining how participant addressed these to continue being active - Praising participant’s handling of lapses to continue activity - Reinforcing positive coping with lapses to avoid relapse - Discussing ways to avoid similar lapses in future |
| Week 6:  Second  Telephone  Session | Motivational Interviewing | - Enhancing rapport by sharing experiences of last three weeks of physical activity - Encouraging discussion of issues by praising activity done, even if limited compared to goals - Exploring feelings of success and achievement to reinforce empowerment - Reflecting on problems and obstacles experienced and encouraging participant to think of ways to resolve problems and surmount obstacles - Reinforcing participant’s positive experience of autonomy in choosing and undertaking own physical activity |
|  | Self-efficacy | - Discussing experiences of physical activity during last three weeks of program - Praising level of activity attained to increase feelings of mastery - Reflecting on degree to which goals set in Week 3 were attained - Encouraging participant to revise goals based on experience   - If goals were easily attained and mastery was experienced, support goals being increased   - If goals were not attained, reflecting on whether they were too high and should be reset to suit currently achievable levels to ensure mastery |
|  | Trans-theoretical Model | - Identifying current stage of change - Praising if participant has moved in a positive direction - Reflecting if stage of change has remained unchanged - Supporting and problem-solving with participant if stage of change has moved in a negative direction - Providing stage-appropriate advice - For example, if participant has remained at action stage, discussing additional behavioral techniques to sustain activity, such as doing activity with a family member or friend to provide mutual support |
|  | Relapse Prevention | - Exploring any experiences of lapsing in activity between Week 3 and Week 6 - Examining how participant addressed these to continue being active - Praising participant’s successful handling of lapses to continue activity - Reinforcing positive coping with lapses to avoid relapse - Discussing ways to avoid similar lapses in future |
